# Toward a New Approach to Cross-Cultural Distinctiveness and Typicality of Human Faces: The Cross-Group Typicality/ Distinctiveness Metric

**DOI:** 10.3389/fpsyg.2019.00124

**Published:** 2019-01-31

**Authors:** Karel Kleisner, Šimon Pokorný, S. Adil Saribay

**Affiliations:** ^1^Department of Philosophy and History of Science, Charles University, Prague, Czechia; ^2^Department of Psychology, Boǧaziçi University, Istanbul, Turkey

**Keywords:** typicality, distinctiveness, geometric morphometrics, cross-culture, face space, morphology

## Abstract

In the present research, we took advantage of geometric morphometrics to propose a data-driven method for estimating the individual degree of facial typicality/distinctiveness for cross-cultural (and other cross-group) comparisons. Looking like a stranger in one’s home culture may be somewhat stressful. The same facial appearance, however, might become advantageous within an outgroup population. To address this fit between facial appearance and cultural setting, we propose a simple measure of distinctiveness/typicality based on position of an individual along the axis connecting the facial averages of two populations under comparison. The more distant a face is from its ingroup population mean toward the outgroup mean the more distinct it is (vis-à-vis the ingroup) and the more it resembles the outgroup standards. We compared this new measure with an alternative measure based on distance from outgroup mean. The new measure showed stronger association with rated facial distinctiveness than distance from outgroup mean. Subsequently, we manipulated facial stimuli to reflect different levels of ingroup-outgroup distinctiveness and tested them in one of the target cultures. Perceivers were able to successfully distinguish outgroup from ingroup faces in a two-alternative forced-choice task. There was also some evidence that this task was harder when the two faces were closer along the axis connecting the facial averages from the two cultures. Future directions and potential applications of our proposed approach are discussed.

## Introduction

Travelers to a foreign country are sometimes mistaken to be local. One of the authors has discovered on his many trips to Turkey as a Czech that he can easily mislead people to believe that he is from the Black Sea region of Turkey. Another of the current authors, during his sabbatical in Prague as a Turk, has frequently found himself being spoken to in Czech by locals who did not realize he is a foreigner. It is possible that such experiences are partly the result of some level of resemblance of our respective faces to the typical outgroup face.

Typicality and the concept of type has traditionally played an important role within all life sciences including psychology, comparative anatomy, and morphology (Galton, 1879, 1883; Russell, 1916; Goethe, 1999; Kleisner, 2007). Typical object, or abstraction of a typical object, results from comparison of many particular occurrences of things. Such typical objects are usually considered as a reference against which all other things in the environment are evaluated. The things perceived as the most distant from a type are realized as distinct and less familiar.

Despite the extensive interest in facial distinctiveness, measuring distinctiveness is somewhat complicated because numerous facial aspects and their interrelatedness determine whether a face is perceived as distinctive (Wickham et al., 2000). In his influential work, Valentine (1991) defined facial distinctiveness as a function of Euclidean distance from populational mean face. In Valentine’s Face Space, the understanding of typical and distinctive faces is not separated and both are covered by the same multidimensional framework. In such multidimensional similarity space, faces are represented as single points in high-dimensional similarity space defined by visual properties (or facial measurements); faces are normally distributed and there is a higher density of faces closer to origin (mean face); typical faces are closer to the origin (mean face) than atypical faces; typical faces are around the mean while atypical faces are on the periphery (Valentine, 1991; Valentine et al., 2016). Valentine’s model is, however, limited to intra-population comparisons since outgroup faces will necessarily form a cluster far away from ingroup mean.

Previous research produced evidence that typicality does affect social perception of faces, focusing mostly on the relationship between face typicality and attractiveness (see, e.g., Langlois and Roggman, 1990; Perrett et al., 1994; Rhodes and Tremewan, 1996; Fink and Penton-Voak, 2002; Rhodes, 2006; DeBruine et al., 2007; Said and Todorov, 2011; Danel et al., 2012; Trujillo et al., 2014). Recently, Sofer et al. (2015) demonstrated that face typicality plays an important role in trustworthiness judgments showing that perceived trustworthiness, but not attractiveness, changes along the cline of facial typicality (Sofer et al., 2015). Moreover, a cross-cultural study on Japanese and Israeli populations revealed that ingroup typical faces were perceived as more trustworthy than outgroup typical faces suggesting that people from different cultures use their own culture-specific typicality cues when judging trustworthiness (Sofer et al., 2017). Furthermore, facial distinctiveness and typicality have been repeatedly shown to be important for face recognition (Bartlett et al., 1984; Valentine, 1991; Valentine and Ferrara, 1991; Vokey and Read, 1992; O’toole et al., 1994; Burton et al., 2005; Valentine et al., 2016). Outgroup perception of typicality is suggested to be the core mechanism of racial stereotypes, where members of a minority that are perceived as more typical (of their own group) face a higher degree of racial prejudice and discrimination (Maddox, 2004; Kahn and Davies, 2011; Hebl et al., 2012). Nevertheless, our work does not focus on how typicality/distinctiveness affects the recognition of individual faces or on the stereotypicality of local minorities. Here we ask to what extent an individual face resembles the standards of ingroup and outgroup population. Our proposed approach is not a refinement of intra-cultural facial typicality/distinctiveness research; rather, it is an extension of it into the area of cross-cultural comparisons.

**FIGURE 1:**
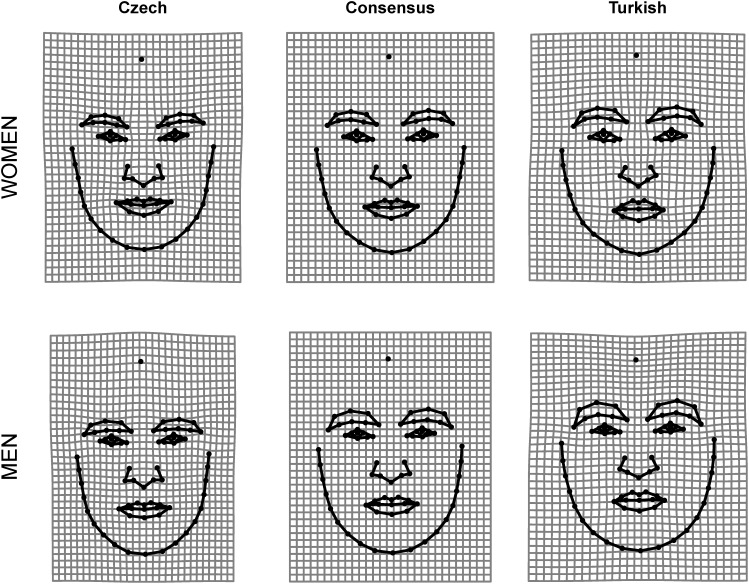
Deformation grids illustrating the differences between Czech and Turkish mean facial shape compared to the global mean configuration. The shape changes were magnified three times for better interpretability.

In this research, we compare faces from two populations, Czech and Turkish that are not closely related but also not extremely distant as to the geographical distance as well as to the distance in similarity space. See Figure 1 for illustration of differences between Czech and Turkish facial morphology. An individual’s face may resemble the standards of facial appearance typical of a foreign population while at the same time being perceived as somewhat distinct within its own population. Therefore, we do not ask how distinct the face is from the facial average of its own population. Rather, the question is how to measure the deviances from morphological standards of own population *toward* the standards of some foreign population. This perspective is crucial if we want to catch the local dynamics of cross-cultural social perception. When a visitor arrives to a foreign country and is encountered by locals, his/her face is not compared to the standards of its home culture but to standards of the local culture, i.e., how his/her face is distinct from the local majority type. In our case, this corresponds to how much a Czech face looks Turkish-like and vice versa.

In general, there are four theoretical options for assessing typicality within a face space: (1) to measure Procrustes distance of all faces from global mean (average of all faces from both cultures under comparison); (2) to calculate distance from each face to local mean of its own population; (3) to calculate distance of each face from mean of outgroup (foreign) population (4) to project individual faces on the axis connecting the local means. Moreover, the objective measures of facial similarity based on Euclidean distances in principal component space has been shown to correspond to the human perception of facial similarity (Tredoux, 2002).

Options 1 and 2 do not allow to determine which of Czech faces are the most similar or dissimilar to Turkish standards and vice versa. The first option reveals only the similarity to a facial average which is the combination of intermediate Czech and Turkish features. The second option only informs about the similarity to local standards but tells nothing about the closeness of face to outgroup mean. For instance, a Czech face that is the most similar to Turks can have the same distance from its local average as the Czech face which is the most dissimilar, because faces are distributed radially to all directions around their local means. The distance from local average remains the best measure of within-culture typicality but does not allow cross-cultural comparison. The third and fourth options are more suitable for cross-cultural comparison because they provide some information about similarity of each face to the outgroup culture. The mean of one population is thus used as a reference to assess the level of distinctiveness of faces from second population and vice versa.

Inspired by research on human sexual dimorphism, we employed computational strategies represented by the third and fourth options that were formerly used for measuring the individual degree of male sex-typicality (Valenzano et al., 2006; Sanchez-Pages and Turiegano, 2010; Komori et al., 2011; Sánchez-Pagés and Turiegano, 2013; Sanchez-Pages et al., 2014; Mitteroecker et al., 2015). Sanchez-Pages et al. (2014) suggested the possibility to use the distance between symmetrized facial configurations of each male individual and average female face as an objective measure of masculinity. In contrast, Mitteroecker et al. (2015) suggested the position of individual male faces along the axis between male and female average shape (maleness shape scores) as a measure of the individual degree of sexual dimorphism. This method, i.e., using two group averages to define an axis of morphological differences were formerly applied also by Komori et al. (2011) and Valenzano et al. (2006). The first approach is computationally identical to the option 3, i.e., distinctiveness measured as Distance from Outgroup Mean (DfOM) while the latter is identical to, option 4, a measure we call here as Cross-Group Typicality/Distinctiveness Metric (CTDM).

The aim of the present research is twofold: Study 1 aims to compare the measure of facial distinctiveness/typicality based on position of an individual face along the vector between ingroup and outgroup mean (CTDM) with a measure based on individual distance from the outgroup mean (DfOM). As the criterion measure, we gathered ratings of how foreign/local various ingroup and outgroup faces look in two cultures. We expect that CTDM will be more tightly correlated with these ratings than DfOM because DfOM does not carry any directional information about face position in morphospace. At the same time, we expect that the difference between correlations (CTDM – typicality/distinctiveness ratings vs. DfOM – typicality/distinctiveness ratings) will be statistically significant.

In Study 2, we used manipulated composites based on different levels of CTDM to estimate the accuracy with which participants categorize outgroup vs. ingroup faces when they are paired. We expect that observers will generally be able to recognize the face of outgroups with accuracy higher than chance. Further, we expect that accuracy in this task will be lower for composite facial pairs showing lower CTDM distance (i.e., when both faces in the pair are closer to their respective outgroup means).

In sum, the main goal of this article is to propose a simple measure of distinctiveness and typicality which could be easily computed and, thanks to its one-dimensional (univariate) continuous nature, used as universal input variable, which reflects the individual degree of typicality/distinctiveness in any cross-group comparison, within all kinds of subsequent statistical modeling. In two studies, we aim to provide preliminary evidence that our new proposed measure behaves in line with theoretical expectations.

## Study 1

### Materials and Methods

In both studies, all procedures followed were in accordance with the ethical standards of the responsible committee on human experimentation and with the Helsinki Declaration. The research (ref. number 06/2017) was approved by The Institutional Review Board of Charles University Faculty of Science. The datasets generated for this study can be found in OSF^1^.

#### Acquisition of Portrait Photographs

We obtained standardized portrait photographs of 100 men (50 Czech, Mean Age ± SD = 23.89 ± 4.0; and 50 Turkish, Mean Age ± SD = 21.54 ± 1.93) and 100 women (50 Czech, Mean Age ± SD = 23.77 ± 4.32; and 50 Turkish, Mean Age ± SD = 21.28 ± 1.34). The participants were instructed to avoid any facial cosmetics and jewelry, seated in front of a white background and asked to pose for the camera with neutral facial expression. The photographs of Czech Targets were taken with a Canon 6D camera using a 85 mm lens, studio flash, and a reflection screen. The distance from the lens to the face of the participant was 1.5 meters. A similar setup was used for collecting photographs of the Turkish targets. For the majority of photographs, a Nikon D90 with a 105 mm lens was used and targets were seated 2.82 meters from the camera (for details see Saribay et al., 2018). The photographs were cropped so that the eyes were in the same absolute height and the same length of neck was visible. The original image files had dimensions that were too large for subsequent online ratings. Therefore, the final image resolution was reduced to 600^∗^745 pixels (width^∗^height).

#### Portrait Ratings

Participants were sent an email inviting them to participate in an online study. A group of 315 Turkish raters (134 men, Mean Age ± SD = 21.13 ± 2.09 and 181 women, Mean Age ± SD = 20.9 ± 1.55) and 123 Czech raters (45 men, Mean Age ± SD = 28.88 ± 12.83 and 78 women, Mean Age ± SD = 27.45 ± 12.56) agreed to participate. Turkish raters were undergraduate students who participated in return for course credit and Czech raters were volunteers. Each rater was shown a total of 100 faces which were a random subset of 100 male and 100 female faces (50 of Turkish and Czech within each gender) in a randomized order, one face at a time. Raters were asked whether each face “looks more like a foreigner or more like a local person?” using a five-point response scale ranging from 1 (*certainly a foreigner)* to 5 (*certainly local*). No other information regarding the stimuli were given. Because higher ratings indicate greater certainty that the rated face belongs to the cultural ingroup, we refer to these ratings as “typicality/distinctiveness” and more specifically as “Turkishness” and “Czechness” when the assessment was done by Turkish and Czech raters, respectively. Inter-rater agreement estimated by Cronbach’s alpha was 0.968 for Turkishness and 0.882 for Czechness. Male and female ratings were highly correlated for both Czechness (*r* = 0.926, *p* < 0.001) and Turkishness (*r* = 0.987, *p* < 0.001).

#### Landmark Digitization and Procrustes Fit

**FIGURE 2:**
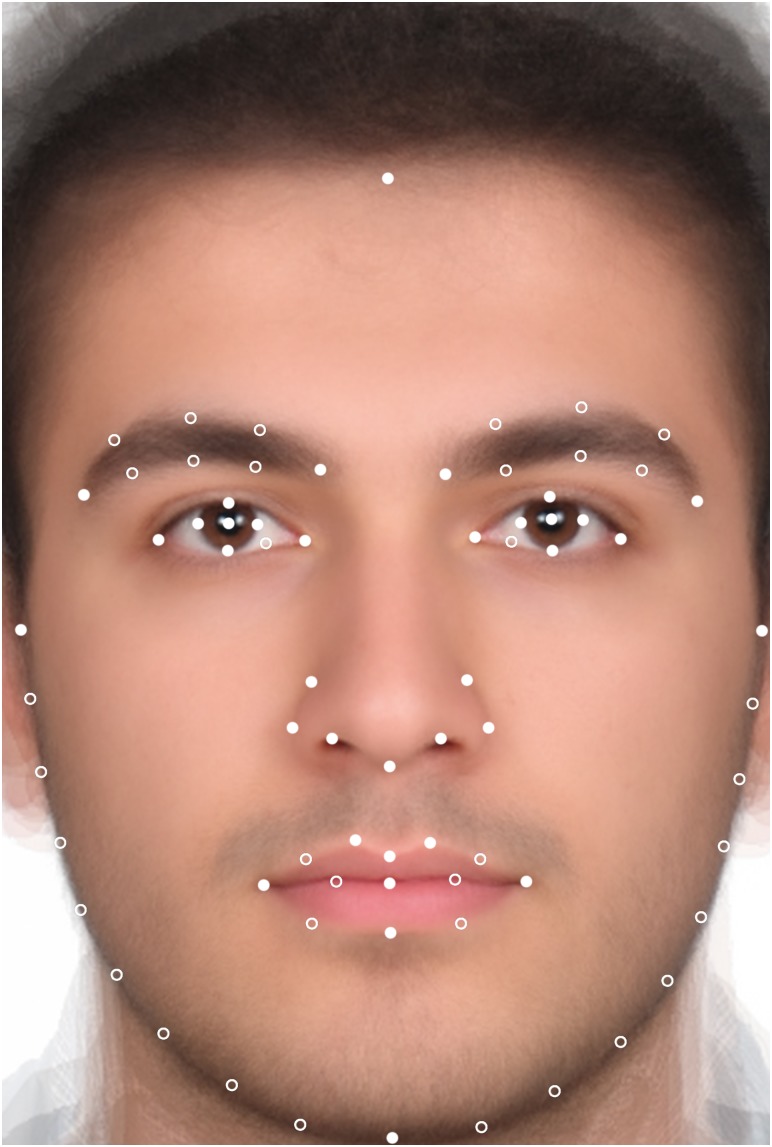
Positions of landmarks and semilandmarks on a face. Landmarks are marked as white filled circles and semilandmarks as empty circles.

Using TpsDig2 software (Rohlf, 2015), we defined 72 landmarks on each face. To specify the shape information along the curves 36 of total number of 72 landmarks were denoted semilandmarks. For definitions and positions of landmarks, see Figure 2 (the same configuration we used in our previous works, e.g., Kleisner et al., 2010; Danel et al., 2016).

All shape data were symmetrized and subsequently subjected to Generalized Procrustes Analysis using the ‘gpagen’ function implemented in the geomorph package in R (Adams and Otárola-Castillo, 2013). We pooled shape coordinates for Czech and Turkish facial configurations and ran GPA analysis on this joined dataset. GPAs were run separately for men and women to avoid shape variation due to sexual dimorphism. This procedure centered, scaled, and rotated all landmark configurations giving aligned shape coordinates (Procrustes residuals). The method that minimizes bending energy between each specimen and the Procrustes mean shape was used to slide the semilandmarks along the curves (Bookstein, 1997). The Procrustes-aligned data were projected to tangent space and used in subsequent multivariate analyses. For purposes of intrasexual comparisons of the two alternative measures of typicality/distinctiveness (DfOM vs. CTDM) the mean Czech and Turkish facial configuration (consensus) was computed separately for male and female faces. The average shape differences between Czech and Turkish facial configuration were visualized using thin plate spline (TPS) deformation grids (Bookstein, 1989; Rohlf and Marcus, 1993; Klingenberg, 2013).

#### Distance From Outgroup Mean (DfOM)

**FIGURE 3:**
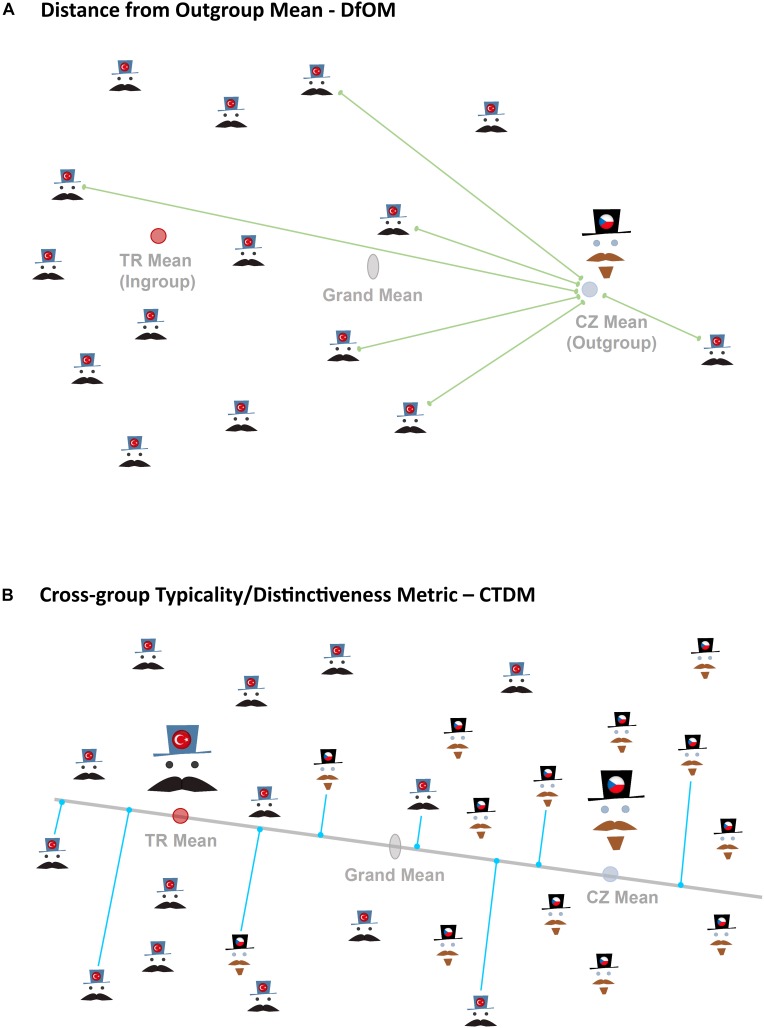
Schematic representation of two approaches to measurement of distinctiveness/typicality compared in Study 1. Upper figure **(A)** demonstrates the approach (DfOM) where the distances of each Turkish face from outgroup mean (Czech average), i.e., green connecting lines, are used as a measure of individual distinctiveness. The shorter the distances (green line) the more distinct the face. Lower depiction **(B)** visualizes the suggested alternative approach (CTDM) taking the orthogonal projection (represented by blue lines) of Czech and Turkish faces on the mean difference vector as a measure of cross-culture distinctiveness. The projection of each face on the mean difference vector can be expressed by scores with negative values in direction to Turkish mean from grand mean and positive values toward the Czech mean. Note that in actual analyses the distances (green lines) or projections (blue lines) of all faces are calculated.

Distance from outgroup mean (DfOM) was computed as the Procrustes distance between the outgroup average facial configuration (consensus) and each face in the set (Figure 3A). The outgroup defined relative to foreign faces at same time represents the ingroup for native faces. Thus the outgroup is understood from the perspective of the visitor to foreign country.

If Turkish faces are compared relative to Czech standards the DfOM is calculated as the distance of all faces (Turkish and Czech) from Czech average and represents a measure that can be compared with ratings of “Czechness.” If Czech faces are compared relative to Turkish standards the DfOM is calculated as the distance of all faces (Turkish and Czech) from Turkish average and represents a measure that can be compared with ratings of “Turkishness.” The shorter is the distance of a face from outgroup consensus the more distinct is the face from its own population and the more typical it is of the foreign population.

#### Cross-Group Typicality/Distinctiveness Metric (CTDM)

To determine the position of an individual facial shape along the axis between ingroup and outgroup mean faces we projected the individual facial configurations in facial morphospace onto the vector between ingroup-outgroup means (see Figure 3B; see also Valenzano et al., 2006; Komori et al., 2011; Mitteroecker et al., 2015). This ‘between-group PCA’ represents a safer alternative to the linear discriminant analysis in cases where the number of individuals do not exceed the number of variables by several times (Mitteroecker and Bookstein, 2011). The position of an individual’s face along the axis connecting Czech and Turkish mean shape corresponds to relative distance of each facial configuration from average Czech and Turkish facial shape. This position can be numerically expressed by scores that correspond to projection of each face onto the principal components of the group averages. These scores vary with changes in facial morphology along the vector intersecting Czech and Turkish means and thus represent a one-dimensional proxy to overall multidimensional facial morphology. Higher negative scores indicate more Czech-like morphology whereas higher positive scores indicate more Turkish-like facial shape.

#### Statistical Analysis

All statistical procedures were performed in R 3.5.0. Kendall rank correlation coefficient was computed to measure the strength of relationship between variables. We compared Kendall’s correlations of CTDM and DfOM with ratings of Turkishness/Czechness (shared variable). The significance of the difference between the correlation coefficients together with their confidence intervals was bootstrapped. 10,000 random populations were sampled from the original data and the expected distribution of the coefficients and the difference between them was calculated. For the purpose of CI estimation, the bonds between ratings, CTDM, and DfOM values within individuals were maintained while sampling the individuals with replacement. This procedure was equivalent to the CI estimation in bootstrap version of “kendall.ci” function in NSM3 package in R (Schneider et al., 2018), which, however, does not take the relationship between more than two variables into consideration. When the distribution of expected differences between correlation coefficient was calculated, CTDM and DfOM vectors stayed unchanged to maintain their correlation within individuals and the ratings of Turkishness/Czechness was sampled with replacement.

To test for the shape differences between Czech and Turkish faces, we employed a permutation test based on comparison of the distance between Czech and Turkish means to the distances obtained by random assignment of observations to these groups. This was done, separately for each gender, by the “permudist” function implemented within the Morpho package in R (Schlager, 2017).

The differences in morphological variation between compared populations may have some effect on cross-cultural discrimination of faces because target faces from less variable population might have been easier to identify as belonging to the ingroup. Therefore, we tested for differences in morphological disparity between Czech and Turkish facial configurations using the function “morphol.disparity” in R’s geomorph package (Adams and Otárola-Castillo, 2013), with significance test based on 9,999 permutations. Using the same routine we also compared the morphological disparity of male and female faces because the facial traits responsible for populational identity might be easier to detect in the less variable gender.

### Results and Discussion

#### Comparison of CTDM and DfOM

As expected, CTDM showed systematically tighter correlations with typicality/distinctiveness ratings (Czechness/Turkishness) than DfOM. The results of Kendall rank correlations (with bootstrapped CIs) are summarized in Table 1. See also Supplementary Table 1 comparing the results of cross-group metrics (CTDM and DfOM) with distance calculated from ingroup means (DfIM). The bootstrap test of difference between Kendall’s correlations revealed that the association of typicality/distinctiveness ratings with CTDM is significantly stronger than with DfOM; this was true for faces of both men (*p* = 0.013) and women (*p* = 0.029).

**Table 1:** Kendall’s rank correlations (with bootstrapped CIs) between Cross-Group Typicality/Distinctiveness Metric (CTDM), Distance from Outgroup Mean (DfOM), and ratings of typicality/distinctiveness by Turkish (Turkishness) and Czech (Czechness) raters.

	Male faces			
	Turkishness		Czechness	
	Kendall’s τ	CIs: 2.5% | 97.5%	Kendall’s τ	CIs: 2.5% | 97.5%
**DfOM**	0.196^∗^	0.067 | 0.321	0.017	-0.113 | 0.147
**CTDM**	0.384^∗∗^	0.271 | 0.488	-0.223^∗^	-0.327 | -0.117
	
	**Female faces**			
	**Turkishness**		**Czechness**	
	**Kendall’s τ**	**CIs: 2.5% | 97.5%**	**Kendall’s τ**	**CIs: 2.5% | 97.5%**

**DfOM**	0.258^∗∗^	0.121 | 0.39	0.111	-0.018 | 0.242
**CTDM**	0.417^∗∗^	0.331 | 0.496	-0.366^∗∗^	-0.473 | -0.254


*See Methods for details on calculation of CTDM and DfOM. Note that CTDM may acquire negative values; higher negative scores indicate more Czech-like morphology whereas higher positive scores indicate more Turkish-like facial shape. ^∗^p < 0.01 and ^∗∗^p < 0.001.*

#### Morphological Differences Between Czech and Turkish Faces

Two group permutation test showed that Czech faces significantly differ in their facial shape from Turkish faces both for faces of men (Procrustes distance between means–PDM = 0.01995, *p* < 0.001) and women (PDM = 0.01634, *p* < 0.001). This means that the members of our target populations differ in average as to their facial structure which makes the cross-group comparison morphologically meaningful.

The analysis of morphological disparity (MD) showed that Czech men (MD = 0.00199) are more variable in facial shape than Turkish men (MD = 0.00154) and Czech women (MD = 0.00182) are more variable in facial shape than Turkish women (MD = 0.00142). These differences were significant for both male (*p* = 0.015) and female (*p* = 0.01) faces. When sex differences were tested, men and women (including both Czech and Turkish faces) did not differ significantly as to the variation in facial shape (*p* = 0.149). These results suggest that Czechs are generally more variable in facial morphology than Turks. The raters might be thus more effective in classifying the Turkish faces as they are (at least in our sample of faces) morphologically more homogeneous. The stronger effects reported for Turkish faces seems to support this expectation.

## Study 2

### Materials and Methods

#### Production of Manipulated Stimuli

We generated manipulated composites in order to estimate the accuracy with which participants categorize a face across different types of trials where the following types of Turkish (TR) and Czech (CZ) face composites are paired: 1: CZ farthest away from TR; 2: Average CZ; 3: CZ closest to TR; 4: TR closest to CZ; 5: Average TR; 6: TR farthest away from CZ (when different types of faces are paired; e.g., 1 vs. 4).

**FIGURE 4:**
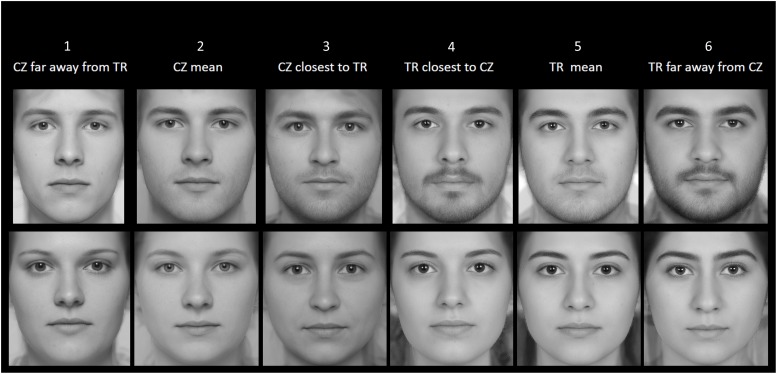
Visualization of manipulated composite stimuli of men (above) and women (below) based on different levels of CTDM along mean difference vector.

The composite faces were generated with use of TpsSuper 2.05 software (Rohlf, 2015). Six facial images of individuals closest to a selected position determined by CTDM value (i.e., CZ farthest away from TR mean; Average CZ; CZ closest to TR mean; TR closest to CZ mean; Average TR; TR farthest away from CZ mean) were used to create composites. See Figure 4 for exposition of manipulated composites and Supplementary Figures 1, 2, men and women, respectively, for the position of composites along CTDM axis.

#### Two-Alternative Forced-Choice (2AFC) Discrimination of Manipulated Stimuli

Participants were sent an email inviting them to participate in an online study. 327 Turkish university undergraduates (153 men, Mean Age ± SD = 20.63 ± 1.46; 171 women, Mean Age ± SD = 20.6 ± 1.8; 3 others, Mean Age ± SD = 23 ± 3.6) participated in return for course credit. Participants were asked to view composite face pairs (whose typicality/distinctiveness was manipulated based on CTDM) and to select the face in each pair that “looks more foreign.” We focused only on cross-group pairs. That is, in each pair, one face belonged objectively to the CZ group and the other to the TR group (with left-right position on the computer screen counterbalanced). This resulted in the following combinations (in terms of the above categories; see *Production of manipulated stimuli* section and Figure 4): 1 vs. 4; 1 vs. 5, 1 vs. 6; 2 vs. 4; 2 vs. 5; 2 vs. 6; 3 vs. 4; 3 vs. 5; 3 vs. 6. One trial for each possible combination was shown to each participant, in random order. Accuracy was defined in terms of whether the face selected as looking more foreign is objectively an outgroup (i.e., Czech) face.

#### Statistical Analysis

All statistical procedures were performed in R 3.5.0. The average difference in proportion of correct responses in trials in which the Czech face was presented on the left vs. right of the screen was -0.0157 (range = -0.059 to 0.036), suggesting that location of faces did not have a systematic effect on responses. Thus, we analyzed the data collapsing across this counterbalancing factor.

##### Overall strategy

We were interested in (1) whether participants were generally able to accurately distinguish outgroup from ingroup faces and (2) whether there would be any difference in the level of this ability in trials with different face pairs. We checked the former using one-proportion z-tests against chance level performance (i.e., 50% accuracy). For the latter, we first conducted Cochran’s Q tests (with the RVAideMemoire package in R; Hervé, 2018) to examine whether any two face pairs differed in the proportion of correct responses that participants produced in those trials. To follow-up the significant Cochran’s Q tests, we conducted a set of selective pairwise comparisons. For each gender (of face), 9 pairs had been presented to participants, resulting in 36 possible pairwise comparisons in total, most of which are theoretically uninteresting or uninterpretable. For ease of interpretation, we focused on comparisons in which we could hold one face constant (e.g., 1_4 vs. 3_4). We selected specific pairs of face pairs to explore the idea that Turkish raters would have more difficulty (i.e., produce lower proportion of correct responses) in trials that involved the Czech face closest to the Turkish average (#3), compared to the Czech faces that are further away from the Turkish average (#1 and #2). That is, the Czech face closest to the Turkish average should be more often mistakenly judged as not being foreign, compared to Czech faces further away from average, holding constant the Turkish face in the pair. We would expect this to be especially true compared to the Czech face furthest away from the Turkish average (#1) and to a lesser extent, to the average Czech face (#2).

This highlights the following 6 comparisons (per gender of face), with the expectation that there would be lower proportion of correct responses in the second pair in each comparison: 1_X vs. 3_X and 2_X vs. 3_X; where X takes the values of 4, 5, and 6 in different trials. We conducted these pairwise comparisons using McNemar’s test (asymptotic; using the rcompanion R package; Mangiafico, 2018), which tests the null hypothesis that the proportion of responses is equal across two face pairs compared. To control for familywise error rate, *p*-values were adjusted using the Hochberg method. We also employed binomial mixed effects models (“glmer” function within lmerTest package; Kuznetsova et al., 2017) with “responses to CTDM trials” (coded as 1 = true answer, 0 = false answer) as a response variable, “type of CTDM trials” as an independent variable, and “raters identity” as a random effect. We have built separate models for male and female facial stimuli. We specified relevant contrasts between trials using “glht” function within multcomp package (Hothorn et al., 2008).

##### Data exclusions

Because the findings could be affected by the presence of raters whose cultural ingroup is other than Turkish, we sought to exclude such raters from the dataset. Participants were asked to report their nationality but this did not capture cultural ingroup-outgroup status or possible differences in amount of exposure to Turkish individuals (e.g., some participants who indicated having a nationality other than Turkish were dual citizens). Instead, we calculated approximately how many months of their lifetime each participant had spent in their homeland by taking the difference between their age in months and the months that they reported being abroad. There were 6 participants who had spent a month or less in Turkey. We excluded these participants from further analyses because their exposure to the Turkish faces appears to be very limited based on this information (1 participant with missing data on this measure was retained).

In the remaining sample, average proportion of correct responses for each participant across the 18 trials of the 2AFC task ranged from 0.16 to 1. There were only 6 participants with an average proportion of correct responses lower than 0.5. The morphological closeness of face pairs presented in the 2AFC trials and hence the difficulty of accurately responding suggests that these low-performers were not necessarily careless or responding randomly. Thus, we retained these low-performing participants. The final sample consisted of 321 participants. These decisions were inconsequential as the findings were not affected by inclusion vs. exclusion of participants on the basis of limited exposure to Turkish faces or low performance in the 2AFC (e.g., the decisions to reject or retain the null hypothesis were unchanged in Table 3).

### Results and Discussion

#### Ability to Distinguish Foreign Faces

We checked whether participants were generally able to distinguish foreign faces from local ones. Table 2 presents the percentage of correct responses in the 2AFC trials across different face pairs separately for male and female target faces. Overall, participants appeared to have no difficulty distinguishing foreign faces. One-proportion *z*-tests indicated that the proportions for all face pairs in Table 2 were significantly greater than chance level (0.5) (*ps* < 0.0001).

**Table 2:** Proportion of correct responses in the 2AFC for each face pair by face gender.

Face pair	Male faces	Female faces
1_4	0.95	0.944
1_5	0.96	0.953
1_6	0.947	0.857
2_4	0.91	0.96
2_5	0.894	0.966
2_6	0.866	0.854
3_4	0.903	0.785
3_5	0.875	0.866
3_6	0.897	0.701
Average within Gender of face	0.911	0.876
Global average	0.894	


*See Figure 4 or the text for the correspondence between values in the face pair column and composite images.*

#### Differences in Ability to Distinguish Foreign Faces Across Face Pairs

Next, we examined whether participant’s ability to produce the correct response (i.e., choosing the outgroup face as “foreign”) differed across face pairs. For this purpose, we conducted a Cochran’s Q test as an omnibus test of whether the proportion of correct responses for any pairs were different within the 9 pairs of male faces and 9 pairs of female faces, separately. The proportion of correct responses differed between at least one comparison within both male, *χ*^2^(8, *N* = 321) = 52.2, *p* < 0.0001; and female face pairs, *χ*^2^(8, *N* = 321) = 235, *p* < 0.0001.

To follow up, we compared selected pairs of face pairs (see *Statistical Analysis* section above) using McNemar’s test. These pairwise comparisons are listed in Table 3. Together with the proportions listed in Table 2, it can be seen that the proportion of correct responses in all significant comparisons are in the expected direction. The comparisons that we expected to be most different are all significant for male faces; whereas the comparisons for which we had less strong expectation of difference are not. For female faces, the comparisons for all pairs were significantly different. The result carried out by binomial mixed effect modeling corroborated the results of the McNemar’s test (see Supplementary Tables 2, 3).

**Table 3:** Pairwise comparisons (McNemar’s Tests) of correct response proportions in trials with different face pairs.

Comparison pair	Male faces		Female faces	
	*χ*^2^	Adjusted p	*χ*^2^	Adjusted p
1_4 vs. 3_4	6.43	0.045*	37.7	<0.001***
2_4 vs. 3_4	0.1	0.752	44.8	<0.001***
1_5 vs. 3_5	18.69	0.000***	18.67	<0.0010***
2_5 vs. 3_5	0.783	0.752	22.26	<0.001***
1_6 vs. 3_6	9.14	0.013*	33.78	<0.001***
2_6 vs. 3_6	3.33	0.204	36.94	<0.001***


*Hochberg-adjusted p-values: ^∗^p < 0.05; ^∗∗^p < 0.01; and ^∗∗∗^p < 0.001.*

There could be limitations arising from the design that could be addressed in future research for a better, confirmatory test of the idea. In sum, while there are apparently other sources of variance in responses in the 2AFC, there is at least preliminary evidence from these results that outgroup faces closer to the ingroup average are more difficult to correctly categorize as “foreign” and/or that outgroup faces further away from the ingroup average are easier to correctly categorize as “foreign.”

## General Discussion

In the present research, we took advantage of geometric morphometrics to propose a sample dependent and thus data-driven method for estimating the individual degree of facial distinctiveness/typicality for cross-cultural comparisons. We attempted to provide preliminary evidence in support of a novel cross-cultural metric of typicality/distinctiveness (CTDM) in two studies. Study 1 revealed significantly tighter association between ratings of typicality/distinctiveness and CTDM than the same ratings and an alternative approach (DfOM). The same pattern was found for faces of both men and women and for both Turkish and Czech raters. The possible weakness of DfOM is that a pure distance measure does not carry information about the position of a face in facial morphospace. The reason why CTDM showed stronger correlations with ratings than DfOM is that DfOM is based on Procrustes distance and can have only positive values. That is, it provides no (or very limited) information about mutual positions of targets (faces) and reference (outgroup mean). The straight lines between individual faces and outgroup mean may form variety of angles with between group axis including right angle. DfOM thus does not account for the fact that faces may theoretically vary in all directions from outgroup mean (see Figure 3A). For instance, two Turkish faces having the same distance from the Czech mean may look dissimilar because one of the faces could be even less similar to Turkish standards than the Czech mean. In contrast, CTDM aligns the positional information of individual faces along the cross-group axis (see Figure 3B). The above-mentioned problematic interpretation of two Turkish faces having the same DfOM despite their apparent dissimilarity thus becomes clearer within the CTDM framework wherein these two faces acquire different CTDM scores.

In Study 2, we assessed accuracy of discrimination of outgroup vs. ingroup composite faces varying along CTDM in a 2AFC task. Performance was generally consistent with our expectations. For instance, when pairs of face pairs were compared, the pairs that contrasted faces closer in CTDM distance (e.g., the Czech face closest to Turkish average and the Turkish face closest to Czech average) were harder to accurately discriminate compared to face pairs in which the two faces are further apart from each other in CTDM. Results involving female (vs. male) faces were more consistent with expectations. This may indicate that female faces carry more information about cultural identity, a possibility that future research could examine.

In hindsight, even though participants showed high overall accuracy, the 2AFC task may have been difficult for participants as the Czech and Turkish faces show only slightly difference in morphology (see Figure 4). Other features of task design may have not been optimal for the current purposes, as well. For instance, because the same faces were repeatedly used across trials, participants may have anchored their responses for repetitions of a given face on their response in the first trial with that face (i.e., consistently judging face 1 to be foreign across trials where it was paired with 4, 5, and 6). This could have masked variability in responses that would otherwise occur. Future tests could better control such irrelevant task features for a purer test of CTDM effects. Despite these limitations, we view Study 2 findings as providing encouraging preliminary evidence for the usefulness of CTDM in predicting performance in ingroup-outgroup face discrimination situations.

### Future Directions, Limitations, and Potential Applications

We used only the shape information for calculation of CTDM but faces are more complex. Hence, the face space can theoretically be augmented with further types of non-shape variables and CTDM may be computed based on this improved face space. Future studies should take into account also facial size, texture, skin color, eye color, hair style and color, contrasts between mouth and surrounding skin, and so on.

Cross-Group Typicality/Distinctiveness Metric can have a broad range of social applications. Previous research showed positive association between judgments of trustworthiness and facial typicality (Sofer et al., 2015, 2017). In cross-cultural interactions, such as bilateral business tradeoffs, student exchange, or even war conflicts, the outgroup individuals closest to the ingroup standards would obtain a substantial advantage because their faces will be more trustworthy-looking to potential, business partners, tutors, or invaders, respectively. In fact, a given face being typical of the ingroup should engender feelings of familiarity, which is known to underlie positive responses to that face (Zebrowitz et al., 2008). Viewed differently, a lack of such typicality and familiarity for an encountered face and/or the face resembling an outgroup prototype is known to make prejudiced reactions toward the face-bearer more likely, sometimes even rendering the situation into a matter of life and death (Blair et al., 2004; Eberhardt et al., 2006). Importantly, these typicality/familiarity-contingent responses to faces may go beyond mere categorization of faces as ingroup vs. outgroup: Perceivers’ responses are often driven by facial cues, not necessarily by the perceived social category of faces (Livingston and Brewer, 2002). The influence of group typicality on social perception had long been ignored with the field of social psychology but has been discussed more explicitly in recent years (Maddox, 2004). However, typicality is often coded in a categorial fashion (i.e., the main distinction being between individuals who are more vs. less typical of a group; e.g., Hebl et al., 2012; Study 2) and/or relying on human judges (e.g., Hebl et al., 2012; Studies 1 and 3). Furthermore, such studies usually focus on only one side of the coin: How typical of the ingroup individuals appear. In the case of research on prejudice against African-Americans in the United States, researchers may implicitly have in mind “smaller distance from White American average” when they mention “low racial stereotypicality” of African-American faces, but this is not made explicit (an African-American face can also be less typical of the ingroup and be closer to a different ethnic group such as Hispanic). CTDM may offer a way to distinguish these “cue- vs. category-based” responses and the influence of individual differences in targets’ appearance in a way that is conceptually clearer, more quantitative and/or objective, and more finely (vs. coarsely) related to face morphology than previous research. A Turkish visitor in Czech Republic should thus receive better treatment from Czech individuals when his/her CTDM is closer to (vs. farther away from) the Czech populational average, in a way that goes beyond him/her being recognized as Turkish (or foreign). In short, CTDM could be used to predict prejudice in intergroup contexts beyond the effect of categorization and could have wide application in social psychology.

Another possible application may link CTDM with face recognition. According to the attractor field model, the object’s similarity and spatial density in multidimensional space is unintuitively interrelated. Face morphs were judged to be more similar to the atypical than to the typical parent image (Bartlett and Tanaka, 1998; Tanaka et al., 1998). It is thus more difficult to detect differences between atypical morphs than differences between typical morphs. This was true for morphs of various inanimate and animate objects such as birds, cars, and faces (Tanaka and Corneille, 2007). There seems to be a tradeoff between spatial density (and higher similarity) of faces around local means and bigger attractor fields around distinctive faces. These distinctive faces thus may be perceived as mutually more similar due to their larger attractor fields than they ‘objectively’ are. Thereof stems one prediction for future research testable with use of CTDM: The individual identity of outgroup faces closest to observers’ ingroup mean should be recognized with higher accuracy than individual identities of faces most distant from observers’ ingroup standards. A Turkish visitor in Czech Republic should thus better recognize the identity of Czech individuals with CTDM values closer to Turkish populational average.

As our research was intended as an initial test of CTDM, there were limitations other than those already discussed. Some differences between images from the two cultural groups need to be better controlled. Even minor differences in technical equipment used to produce images (e.g., focal length of the camera lens) could result in different images (Třebický et al., 2016). Other stylistic differences such as facial hair may also influence ratings. Future research should seek to remedy these problems.

### Further Theoretical Considerations: A Note on the Limitations of Composite Images in Face Research

Portrait photograph blending is an old procedure, first used by Francis Galton who applied it to reveal features typical of certain categories of people (Galton, 1879, 1883). A long time since Galton, average composites are still used, with much technical improvement. Many researchers use manipulated composite stimuli to investigate various causative effects of facial traits on social impressions. We see this research agenda as at least partially problematic (see also Schaefer et al., 2009; Jones, 2018; Windhager et al., 2018). Calibrating facial morphs for use as stimuli in biological studies of social perception. Scientific reports, 8(1), 6698. doi: 10.1038/s41598-018-24911-0. Unlike individual images, composites yield clear results even when the sample size is low. For instance, Rhodes (2006) reported a strong positive relationship between symmetry and attractiveness in composite faces, but only moderate effects in non-manipulated faces (Rhodes, 2006). Moreover, recent cross-cultural evidence showed only moderate or no relationship between attractiveness ratings based on non-manipulated facial photos and averageness computed as each face’s distance from sample Procrustes mean (Kleisner et al., 2017). The use of experimentally manipulated stimuli thus has various practical consequences, such as greater effect sizes and a higher probability of positive results. Moreover, stimuli experimentally manipulated for a particular research purpose become a reification of researchers’ theoretical needs. This is a problem when such stimuli are not used as research tools but substituted for natural objects (faces), that is, when individual faces with their natural variations are substituted for manipulated stimuli whose variation is constrained in a way that *a priori* corresponds to expectations given by a theory. By constraining the variation of stimuli, we also limit the variation of possible responses to these stimuli. One might claim that this is how experimental science works, which may well be so, but properties of the experimental toolkit must be included in the interpretation of the results. During twentieth century, this condition has been widely discussed within philosophy of science and become an indispensable part of some fields of experimental physics. Yet, it remains largely neglected in evolutionary psychology and biology.

What is the alternative? First, to use non-manipulated stimuli. Second, to use stimuli manipulated so as to correspond to the observed range of natural variation. Third, to use both manipulated and non-manipulated stimuli; the difference in results could then be used as a background to the overall interpretation of results. This can be easily accomplished by application of geometric morphometrics and related multivariate techniques, such as in the CTDM approach presented here, that provide direct statistical control over the direct stimuli analysis and their manipulation.

## Conclusion

To conclude, distinctiveness and typicality are two sides of the same coin so claiming that a face is either more/less distinct or more/less typical depends on which populations are taken as ingroup and outgroup. CTDM allows one to estimate the degree to which an individual from a given (ingroup/local) population resembles the facial standards of another (outgroup/foreign) population and vice versa. When mathematically expressed, such knowledge is potentially useful for studying relationships between the individuals’ degree of cultural distinctiveness/typicality perceived by others and attributions of attractiveness and personality traits across cultures. Further, CTDM allows us to generate manipulated stimuli that respect the natural variation of human faces within a particular population. Finally, CTDM is not constrained to human faces and can be applied to any shape such as parts of the human body and cultural artifacts. We hope that future research will provide further evidence of CTDM’s utility and realize its potential for application in face research and beyond.

## Ethics Statement

This study was carried out in accordance with the recommendations of “The Institutional Review Board of Charles University Faculty of Science” with written informed consent from all subjects. All subjects gave written informed consent in accordance with the Declaration of Helsinki. The protocol was approved by the “The Institutional Review Board of Charles University Faculty of Science” (protocol ref. number: 06/2017).

## Author Contributions

KK and SAS developed the study concept and drafted the manuscript. Data collection was performed by SAS in Turkey and by ŠP and KK in Czechia. All authors performed the data analysis and result interpretation, provided critical revisions, and approved the final version of the manuscript for submission.

## Conflict of Interest Statement

The authors declare that the research was conducted in the absence of any commercial or financial relationships that could be construed as a potential conflict of interest.

## Footnotes

1 https://osf.io/wh7mf/
