# Supplementary material for: Toward a New Approach to Cross-Cultural Distinctiveness and Typicality of Human Faces: The Cross-Group Typicality/ Distinctiveness Metric
Source: Front Psychol. 2019 Jan 31;10:124. doi: 10.3389/fpsyg.2019.00124 (PMC6365443; doi:10.3389/fpsyg.2019.00124)
Supplement: Supplementary file 1 [file Table_1.DOCX]

| Suplementary table 1. Kendall's rank correlations (with boostrapped CIs) between Cross-Group Typicality/Distinctiveness Metric (CTDM), Distance from Outgroup Mean (DfOM), Distance from Ingroup Mean (DfIM), and ratings of typicality/distinctiveness by Turkish (Turkishness) and Czech (Czechness) raters. |
| --- |

|  | **Male Faces** | | | |
| --- | --- | --- | --- | --- |
|  | **Turkishness** | | **Czechness** | |
|  | Kendall's τ | CIs: 2.5% \| 97.5% | Kendall's τ | CIs: 2.5% \| 97.5% |
| **DfOM** | 0.196** | 0.067 \| 0.321 | 0.017 | -0.113 \| 0.147 |
| **DfIM** | -0.047 | -0.177 \| 0.085 | 0.06 | -0.069 \| 0.190 |
| **CTDM** | 0.384*** | 0.271 \| 0.488 | -0.223** | -0.327 \| -0.117 |
|  | **Female Faces** | | | |
|  | **Turkishness** | | **Czechness** | |
|  | Kendall's τ | CIs: 2.5% \| 97.5% | Kendall's τ | CIs: 2.5% \| 97.5% |
| **DfOM** | 0.258*** | 0.121 \| 0.39 | 0.111 | -0.018 \| 0.242 |
| **DfIM** | -0.085 | -0.228 \| 0.061 | -0.033 | -0.170 \| 0.102 |
| **CTDM** | 0.417*** | 0.331 \| 0.496 | -0.366*** | -0.473 \| -0.254 |

Supplementary table 2. Summary of the results of binomial mixed effects modeling from Study 2.

| **Men** | **Random effects** | **Variance** | **SD** |  |  |
| --- | --- | --- | --- | --- | --- |
|  | Rater’s identity | 7.463 | 2.732 |  |  |
|  | **Fixed effects** | **Estimate** | **SE** | **z-value** | **p-value** |
|  | Intercept | 5.528 | 0.506 | 10.918 | < 0.001*** |
|  | 1_5 | 0.199 | 0.458 | 0.436 | 0.663 |
|  | 1_6 | -0.196 | 0.434 | -0.452 | 0.651 |
|  | 2_4 | -0.998 | 0.401 | -2.487 | 0.013* |
|  | 2_5 | -1.332 | 0.393 | -3.394 | <0.001*** |
|  | 2_6 | -1.811 | 0.384 | -4.715 | <0.001*** |
|  | 3_4 | -1.279 | 0.394 | -3.248 | 0.001 ** |
|  | 3_5 | -1.673 | 0.386 | -4.333 | <0.001*** |
|  | 3_6 | -1.325 | 0.393 | -3.373 | <0.001*** |
| **Women** | **Random effects** | **Variance** | **SD** |  |  |
|  | Rater’s identity | 3.04 | 1.743 |  |  |
|  | **Fixed effects** | **Estimate** | **SE** | **z-value** | **p-value** |
|  | Intercept | 3.971 | 0.314 | 12.646 | <0.001*** |
|  | 1_5 | 0.151 | 0.388 | 0.390 | 0.697 |
|  | 1_6 | -1.393 | 0.326 | -4.268 | <0.001*** |
|  | 2_4 | 0.234 | 0.394 | 0.593 | 0.553 |
|  | 2_5 | 0.615 | 0.426 | 1.445 | 0.149 |
|  | 2_6 | -1.398 | 0.326 | -4.284 | <0.001*** |
|  | 3_4 | -2.014 | 0.318 | -6.329 | <0.001*** |
|  | 3_5 | -1.256 | 0.329 | -3.816 | <0.001*** |
|  | 3_6 | -2.634 | 0.316 | -8.339 | <0.001*** |

Supplementary table 3. Summary of the comparison between pairs of trials from Study 2 based on results from binomial mixed effects modeling.

| Comparison Pair | Male Faces | | | Female Faces | | |
| --- | --- | --- | --- | --- | --- | --- |
|  | *Estimate ±SE* | *z value* | *p value* | *Estimate ±SE* | *z value* | *p value* |
| 1_4 vs. 3_4 | 1.2793 ±0.3938 | 3.248 | 0.007** | 2.0137 ±0.3182 | 6.329 | <0.001 *** |
| 2_4 vs. 3_4 | 0.2813 ±0.3369 | 0.835 | 0.947 | 2.2472 ±0.3376 | 6.656 | <0.001 *** |
| 1_5 vs. 3_5 | 1.8725 ±0.4024 | 4.653 | <0.001 *** | 1.4073 ±0.3408 | 4.130 | <0.001 *** |
| 2_5 vs. 3_5 | 0.3405 ±0.3120 | 1.091 | 0.838 | 1.8710 ±0.3842 | 4.870 | <0.001 *** |
| 1_6 vs. 3_6 | 1.1287 ±0.3789 | 2.979 | 0.017 * | 1.2406 ±0.2391 | 5.190 | <0.001 *** |
| 2_6 vs. 3_6 | 0.4861 ±0.3087 | -1.574 | 0.5 | 1.2357 ±0.2389 | 5.173 | <0.001 *** |

Significance levels: *p < .05 **p<.01 ***p<.001

Supplementary figure 1: Histogram plot showing distribution of CTDM values for faces of men. The vertical lines stand for the average CTDM values of composites marked by respective numbers. See Figure 4 for exposition of manipulated composites (1-6).

Supplementary figure 2: Histogram plot showing distribution of CTDM values for faces of women. The vertical lines stand for the average CTDM values of composites marked by respective numbers. See Figure 4 for exposition of manipulated composites (1-6).
